# An Eco-Friendly Antheraea Pernyi Silk Gland Protein/Sodium Alginate Multiple Network Hydrogel as Potential Drug Release Systems

**DOI:** 10.3390/gels9010004

**Published:** 2022-12-22

**Authors:** Jia Li, Bo-Xiang Wang, De-Hong Cheng, Yue Zhang, Qiang Yao, Xin-bin Ji, Jing Guo, Yan-Hua Lu

**Affiliations:** 1 School of Textile and Material Engineering, Dalian Polytechnic University, Dalian 116034, China; 2 Liaoning Provincial Key Laboratory of Functional Textile Materials, Liaodong University, Dandong 118000, China

**Keywords:** antheraea pernyi silk gland protein, sodium alginate, multiple network hydrogel, tissue engineering, drug release systems

## Abstract

To improve the versatility of the sodium alginate-loaded bio-hydrogels, Antheraea pernyi silk gland protein/sodium alginate drug-loaded hydrogels were prepared by using an eco-friendly multiple network cross-link technology. Fourier transform infrared (FT-IR) spectroscopy and UV-Vis spectrophotometer were used separately to evaluate the chemical structure and drug release behavior of drug-loaded hydrogels. The antibacterial drug carrier gels were evaluated by using inhibition zone test against the *S. aureus* and *E. coli*. The CCK-8 assay was used to assess the biocompatibility of drug loaded hydrogels. The FT-IR results showed that there was a strong interaction within the drug loaded hydrogels, and the ASGP was beneficial to enhance the interaction within the drug loaded hydrogels. UV-Vis spectrophotometer results indicated the cumulative release reached 80% within 400 min. Antibacterial bio-hydrogels had a good antibacterial property, especially the antibacterial bio-hydrogels with bacitracin exhibits superior to other antibacterial agents. The drug-loaded bio-hydrogels exhibited the adhesion and proliferation of RSC96 cells and perfected biocompatibility. This provides a new idea for further research and development of tissue-friendly drug-loaded biomaterials.

## 1. Introduction

Bio-hydrogels have become one of the most potential materials, especially drug-loaded bio-hydrogels in the field drug delivery, cell encapsulation, tissue repair, reconstructive surgery, and other biological application [1,2,3]. Owing to their eco-friendly, biocompatibility, and non-toxicity to the human body, natural materials are extensively studied for bio-hydrogel fabrication. Among them, silk protein and sodium alginate have been used in the biological applications including tissue engineering and drug delivery [4,5,6]. In recent times, hydrogels based on silk protein and sodium alginate are extensively investigated and utilized [7,8,9,10]. The reported the wound dressings of SF/Rhein hydrogels could enhance the healing rate of burn wounds, expediting angiogenesis, and promoting skin appendages formation [11]. The novel SF-SANPs had the ability to load and control the release rate of VANCO in a pH-sensitive manner [12]. Although tussah silk fibroin is concerned by many researchers for tissue regeneration of bone, cartilage, skin, blood vessels, nerve, and other biological application, the traditional extractive technique of tussah silk protein is usually using tussah cocoon as raw material and the process is complicated and pollutes the environment [13,14]. However, the structure of silk protein is seriously damaged, and the result molecular weight of silk protein is low by these approaches [15,16].

The fifth instar larvae of antheraea pernyi silk gland protein with no physiological toxicity, produces cell adhesion and growth catch the eye of researchers, which expect to be one of the important research objects for biological materials and applied to the drug slow-release, wound healing and tissue repair [17,18,19]. The combine of Antheraea pernyi silk gland protein and sodium alginate as a natural biological material is desirable for improving the poor formability of tussah silk gland protein and giving water gel good hydrophilicity and flexibility [20,21,22,23,24]. However, the research of new biomedical materials based on silk grand protein of antheraea pernyi is still in its infancy [25]. It is significant research value and application prospect that taking the fifth instar larvae of antheraea pernyi as the research object. In a previous study [26], we have investigated the possibility of Antheraea Pernyi silk gland protein/sodium alginate drug-loaded hydrogels with cell and drug delivery by composite coagulation bath including citric acid solution and CaCl_2_ solution. In above paper, the bacteriostatic activity of bio-hydrogels was not investigated. In addition, in the application of hydrogels materials, hydrogels not only bring convenience to clinical treatment, but also infection caused by biological materials is a very serious problem in the current clinical use [27]. Microbial infection is one of the most common complications in clinical medicine that limited the wide application of biomaterials [28,29]. Thus, on the basis of the former critics’ research, this study attempts to explore the possibility of the Antheraea pernyi silk gland protein/sodium alginate drug-loading bio-hydrogels with antibacterial properties by single coagulation bath (citric acid solution). Further, compared with the traditional silk protein regeneration extraction technology, this method uses live fifth instar tussah silkworm as raw material and natural antibacterial material as antibacterial agent. The production process is simple and pollution-free. We abandon the environmental pollution and the damage of the macromolecular structure of silk protein caused by the traditional complex extraction process. We aim to promote the use of natural green environmental protection natural Antheraea pernyi silk gland protein to prepare antimicrobial drug-loaded bio-hydrogel, which is expected to be used in various biotechnology and tissue engineering fields.

## 2. Results and Discussion

### 2.1. Synthesis Drug Loaded Hydrogels

**Figure 1:**
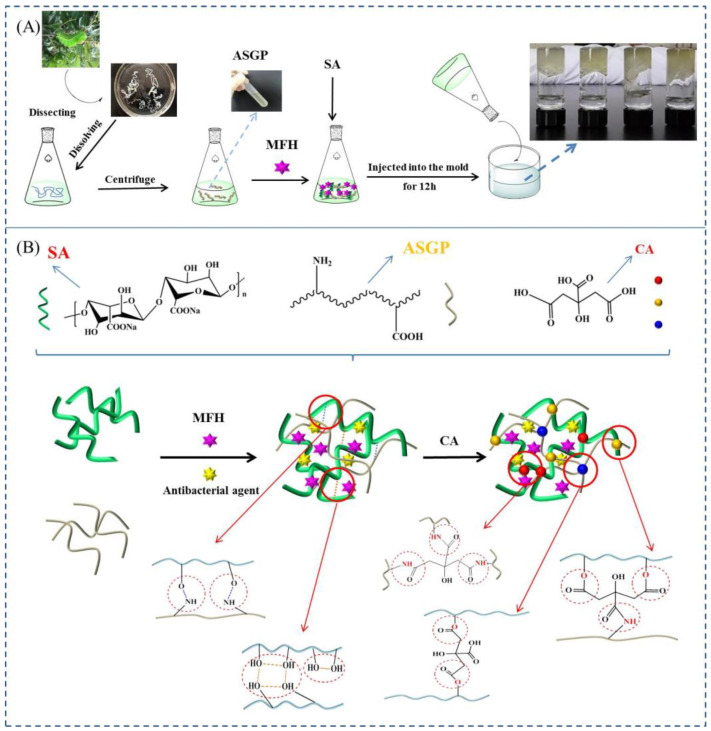
(**A**) Preparation of drug loaded hydrogels; (**B**) A schematic illustration of the synthesis of drug loaded hydrogels.

The citric acid as curing agent for sodium alginate and silk protein has been extensively researched [30,31,32,33]. SA and ASGP contains a large number of hydrogen bond donors and acceptors, such as nonpolar amino acids, hydrophobic amino acids, and polar amino acids [34,35]. Therefore, the ASGP-SA antibacterial drug loaded multi-network bio-hydrogels integrating “covalent bond-ionic bond-hydrogen bond” was prepared by a simple curing method. The schematic illustration and mechanism of antibacterial drug loaded multi-network bio-hydrogels is shown in Figure 1.

The first network hydrogels can be prepared by blending SA into ASGP solution according to the different proportion distribution and joining MFH and antibacterial agents at the same time. The first network structure with lots of hydrogen bonds can be formed between SA and ASGP and among SA. Then, the above ASGP-SA network hydrogels were dumped into a mold with citric acid curing agent. Multiple hydrogen bonds (ASGP-CA, SA-CA, ASGP-SA-CA) network structures can be formed between ASGP, SA, and CA (shown in Figure 1B). It was assumed that, first, the forming of the hydrogen bond between the –OH of SA and –NH_2_ of ASGP, or among the –OH of SA; second, the nucleophilic substitution occurred between car-boxyl groups in citric acid and amine groups in ASGP or –OH in SA as nucleophiles; with average more than one carboxyl group in one citric acid participated in the reaction with one or more ASGP or SA molecules, the ASGP was intra- or inter-crosslinked [36,37]. The reaction was occurred between the hydroxyl of SA, the amino acid of ASGP and the carboxyl of CA formed bond ionic. 

**Figure 2:**
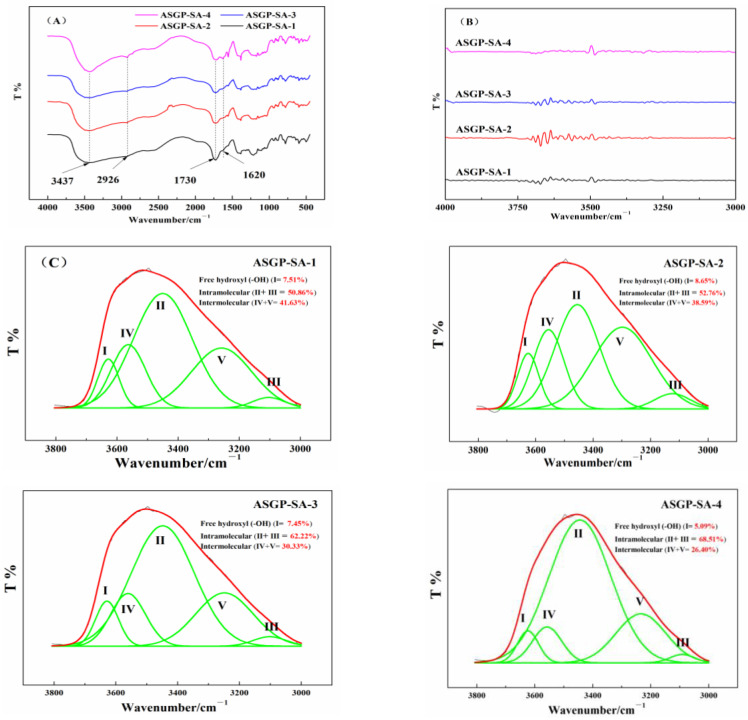
(**A**) FT-IR spectra, (**B**) the second derivative spectra, and (**C**) gauss fitting curves of drug loaded hydrogels.

The FT-IR spectrums, the second derivative spectra and gauss fitting curves of drug loaded hydrogels were shown in Figure 2. The hydrogen-bond interaction of SA, ASGP, and CA in the drug loaded hydrogels was studied by the FT-IR second-derivative spectra being smoothed, then treated with Gauss peak fitting of peak analyzer ranging from 3000 to 3800 cm^−1^ (Figure 2C). 

In previous studies, 3437 cm^−1^ is –OH vibrations, 2926 cm^−1^ is –CH of six-membered ring vibrations, 1730 cm^−1^ is –COO– vibrations, and 1620 cm^−1^ is >C=O bending [38]. In this study, for the FT-IR spectra of drug loaded hydrogels (Figure 2A,B), the characteristic signals of SA, ASGP, and the drug loaded hydrogels appeared at 3437 cm^−1^, 2926 cm^−1^, 1730 cm^−1^, and 1620 cm^−1^, respectively, which were related to –OH stretching vibration, –CH of six-membered ring stretching vibration, –C=O (carboxyl and ester) confirming the curing formation, and –COO– asymmetric stretching vibration, respectively. The above results were coincided with the results of the literature. The above result has also confirmed the curing formation with the FT-IR spectra for drug loaded hydrogels. Different types of hydrogen bond interactions between SA, ASGP and CA in the drug loaded hydrogels are shown in Figure 2C. It can be seen from Figure 2C that the hydrogen bonds between drug loaded hydrogels mainly include free hydroxyl groups, intramolecular hydrogen bonds and intermolecular hydrogen bonds. It can be found that the contents of free hydroxyl, intramolecular hydrogen bond, and intermolecular hydrogen bond of ASGP-SA-1 with the peak area after fitting are 7.51%, 50.86%, and 41.63%, respectively. With the increase in the content of ASGP, the contents of free hydroxyl and intermolecular hydrogen bond decrease to 5.09% and 26.40, respectively. However, the intramolecular hydrogen bond content increased to 68.51%. The results indicates that there was a strong interaction within the drug loaded hydrogels, and the ASGP was beneficial to enhance the interaction within the drug loaded hydrogels [39]. The more content of intramolecular hydrogen bond, the better complete expanded in the release medium for drug loaded hydrogels.

### 2.2. Morphological Observation

**Figure 3:**
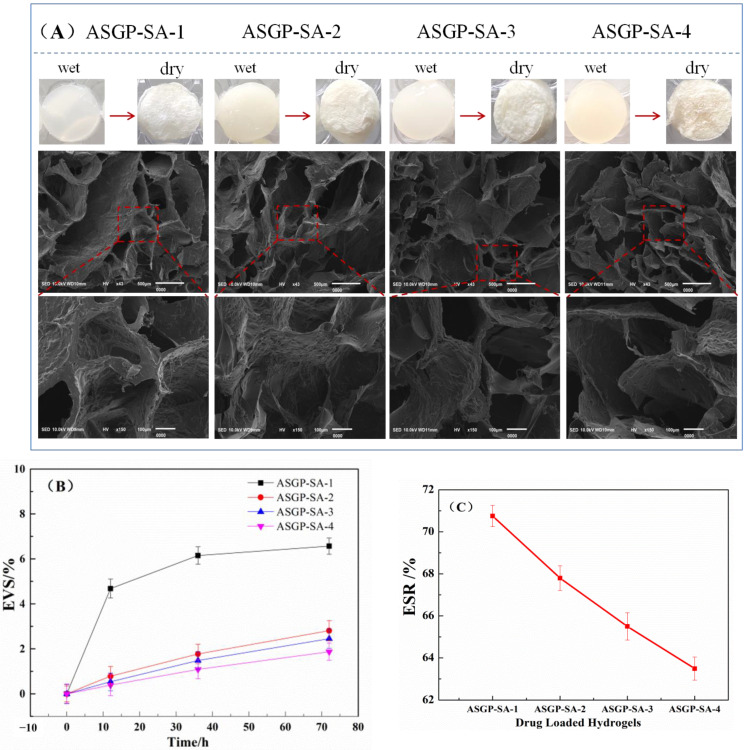
(**A**) SEM, (**B**) stability, and (**C**) swelling ratio of drug loaded hydrogels.

The wet drug loaded hydrogels were appeared opalescent and opaque. With the increase in ASGP, the color of wet drug loaded hydrogels deepened, and was light yellow. The dry drug loaded hydrogels were obtained after freeze-drying. The apparent morphology of the dry drug loaded hydrogels was observed by SEM. The test results are shown in Figure 3A. It can be seen from Figure 3A that drug loaded hydrogels with different mixed proportion all have the uniform porous structures which is due to the water sublimation and intermolecular and intramolecular interactions among polymers in the loaded hydrogels during freeze-drying [40,41]. The more protein content of ASGP, the more pore structure will be uniform and the denser structure of hydrogels. As the proportion of ASGP increases, the interaction between ASGP and SA increases. The structure of the drug-loaded gel polymer is compact, and the regular porous structure is gradually formed during freeze drying. The results were consistent with those of swelling ratio and volume stability of the drug loaded hydrogels.

The volume stability test results of all drugs loaded hydrogels are shown in Figure 3B. As can be seen from the Figure 3B, the volume changes of ASGP-SA-1 in the release medium was significantly, EVS was 0 to 6%. In addition, the EVS of drug loaded hydrogels was decreased with the increasing content of ASGP. The drug-loading hydrogels showed well volume stability. According to the results of ESR test (Figure 3C), the swelling rate of the drug-loaded hydrogels was the lowest with the increasing content of ASGP, and the swelling rate was 63%. The stability of the drug-loaded hydrogels was consistent with the ESR of the drug-loaded hydrogels. This may be because the more intermolecular hydrogen bond and the littler intramolecular hydrogen bond of the drug-loading hydrogels with the increasing of ASGP. Thus, the reducing ESR of the drug-loading hydrogels is conducive to the stability of the drug-loading hydrogels.

### 2.3. In Vitro Drug Release of Drug Loaded Hydrogels

**Figure 4:**
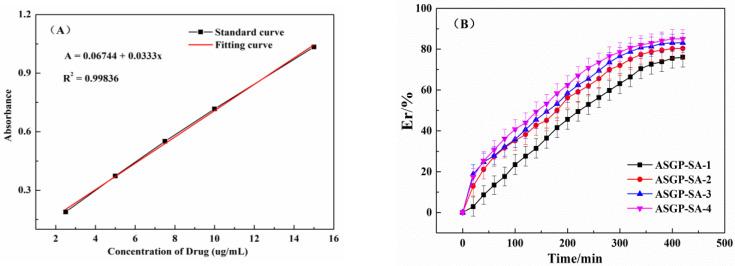
(**A**) Standard curve of MFH, (**B**) the drug release performance of drug loaded hydrogels.

The drug release of the drug loaded hydrogels was analyzed to take simulated intestinal fluid as the drug release medium of the drug loaded hydrogels. The MFH release standard curve and cumulative drug releases of different treatment time of the drug loaded hydrogels were shown in Figure 4A,B. The figure shows that the cumulative liquid gel drug release quantity all can reach 70%. This is due to large amounts polar groups of the drug loaded hydrogels, such as hydroxyl, carboxyl, and amino, which can form hydrogen bonds. In addition, the more ASGP, the more drugs cumulative releases which can reach 80% drugs cumulative releases. With the increase in ASGP content, intermolecular hydrogen bond and intermolecular interaction force of drug loaded hydrogels increased. This is because drug loaded hydrogels can be completely expanded in the release medium, the molecular chain of ASGP can be completely free and easy to form random curl, which is conducive to drug release of drug loaded hydrogels [42,43]. In addition, the carboxyl group of SA can be deprotonated. And the electrostatic repulsion of free carboxyl ions is conducive to the prolongation of SA molecular chain, at which time the drug is released by osmotic diffusion. The intramolecular hydrogen bond is beneficial to molecular chain free and forming random curl for ASGP-SA, which is correspond to the results of in vitro drug release of drug loaded hydrogels.

The drug loading (D_L_) and encapsulation rate (E_N_) of the drug loaded hydrogels were shown in Table 1.

As can be seen from Table 1, the Er of the drug loaded hydrogels ranges from 70% to 73%, and the D_L_ ranges from 400 mg/g to 450 mg/g. The drug loaded hydrogels have a good embedding effect on MFH. Er represents the ratio of the amount of encapsulated drug in the system to the total amount of drug in the system, and the amount of loaded drug represents the ratio of the amount of drug contained in the hydrogel to the mass of the hydrogel. In order to investigate the release mechanism of MFH, the diffusion kinetics of MFH in composite hydrogels was measured. The First order (1), Higuchi model (2), and Korsmeyer-Peppas model (3) [44,45,46] empirical equation was fit for the drug cumulative release percentage:

*M_t_*/*M*_∞_ = 1 − e^−kt^ (1)

*M_t_*/*M*_∞_ = kt^1/2^ (2)

*M_t_*/*M*_∞_ = kt*^n^* (3)

where *M_t_*/*M*_∞_ is the percentage of drug released at time t the time point, k is the apparent drug release rate constant, *n* is the drug diffusion coefficient.

**Figure 5:**
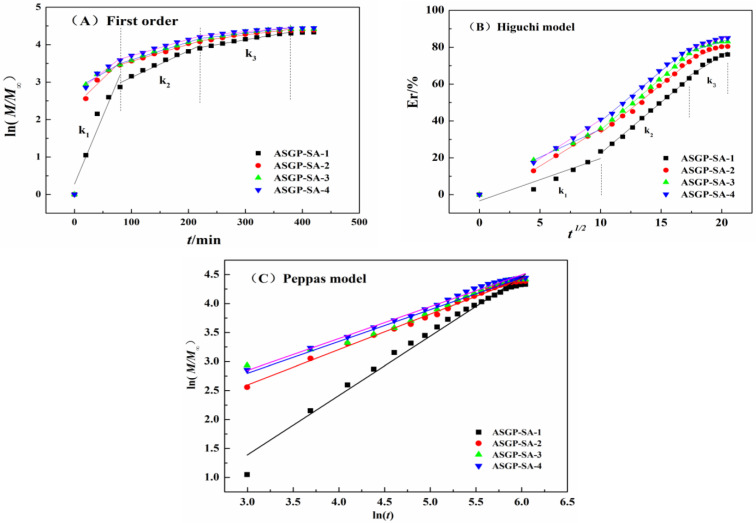
The fitting curve of antimicrobial drug-loaded bio-hydrogels (**A**) First order, (**B**) Higuchi model, and (**C**) Peppas model.

*n* can be used to judge the drug release mechanism. Where, *n* is equal to the slope of the curve with *ln*(*M_t_*/*M*_∞_) as the ordinate and *ln*t as the abscissa, as shown in Figure 5C, is indicated when *n* < 0.43, drug release follows Fickian diffusion; 0.43 < *n* < 0.85, the drug release mechanism was irregular diffusion, that is, both drug diffusion and polymer relaxation controlled the drug release process; *n* > 0.85, drug release follows the Case-II mechanism, that is, drug diffusion is only affected by polymer relaxation or gel degradation during swelling [47,48]. 

The fitting results of the dynamic model are shown in the Figure 5 and Table 2. For first order model (Figure 5A); the drug release of different antimicrobial drug-loaded bio-hydrogels is divided into three stages (k_2_ > k_3_ > k_1_); and the drug release mainly occurs from 100 min to 250 min; at which time, there is no significant change in MFH drug release velocity of different samples. For Higuchi model (Figure 5B), the drug release of different drug loading gel was divided into three stages, and the k value of the three stages did not change significantly, which indicates that MFH showed continuous release, and MFH drug release velocity of different samples did not change significantly at this time.

As can be seen from Figure 5C, drug diffusion coefficients of different antimicrobial drug-loaded bio-hydrogels were all more than 0.43 (*n*_1_ = 1.023, *n*_2_ = 0.610, *n*_3_ = 0.548, and *n*_4_ = 0.546). For ASGP-SA-1, the drug diffusion coefficient (*n*_1_) was greater than 0.85, which indicated that the drug diffusion followed the Case-II mechanism. That is, drug diffusion was only affected by drug-loaded bio-hydrogels relaxation during swelling. With the increasing of ASGP (ASGP-SA-2, ASGP-SA-3, and ASGP-SA-4), drug diffusion coefficient (*n*_2_, *n*_3_, and *n*_4_) were between 0.43 and 0.85, which indicated that drug diffusion followed Fickian diffusion. The drug release of bio-hydrogels was controlled by the drug diffusion and polymers relaxation. The drug on the surface of bio-hydrogels began to diffuse to the outside, and the hydrogel becomes loose to promote the entry of the solution after the bio-hydrogel was immersed in the SIF. MFH was diffused and released to the outside under the action of osmotic pressure that was in line with the Fickian diffusion model during the release.

In conclusion, the Peppas model equation has the best fit among all the kinetic models, and the release mechanism of different drug loading gel can be expressed by the Peppas model release model, with the fitting coefficient R^2^ > 0.98.

### 2.4. Anti-Bacterial Activity Assay

**Figure 6:**
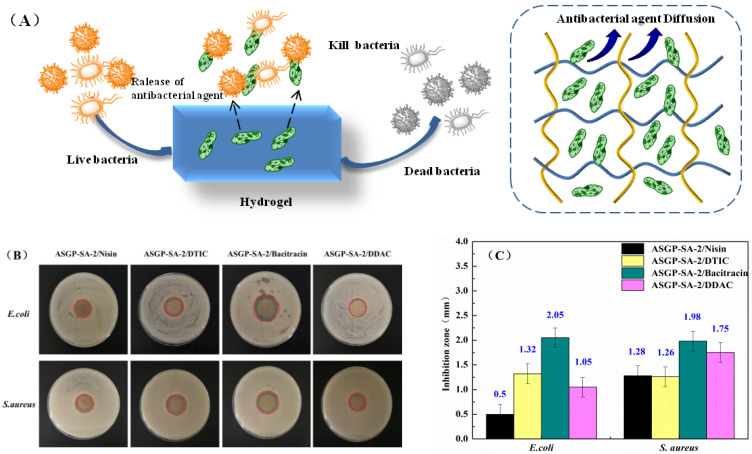
(**A**) Diffusion and release, (**B**) the anti-bacterial activity, and (**C**) the inhibition zone (*n* = 3) against different bacteria species of the ASGP-SA-2/Nisin, ASGP-SA-2/DTIC, ASGP-SA-2/Bacitracin, and ASGP-SA-2/DDAC.

The anti-bacterial activity of ASGP-SA-2/Nisin, ASGP-SA-2/DTIC, ASGP-SA-2/Bacitracin, and ASGP-SA-2/DDAC was shown in Figure 6. As Figure 6A,B illustrate, all the samples had the anti-bacterial activity. The best anti-bacterial activity of ASGP-SA-2/Bacitracin was observed against *E. coli* (2.05 ± 0.2 mm) and *S. aureus* (1.98 ± 0.2 mm). This observation can be attributed to bacitracin which destroys the cell membrane of bacteria, produces perforation and results in the overflow of cell contents and death. In addition, the anti-bacterial activity of ASGP-SA-2/Nisin, ASGP-SA-2/DTIC, and ASGP-SA-2/DDAC was similar. However, ASGP-SA-2/Nisin created a 1.28 ± 2 mm inhibition zone against *S. aureus*, which higher than that of *E. coli* (0.5 ± 2 mm) (Figure 6C). This is because Nisin is a hydrophobic and positively charged small peptide that can be adsorbed on the cell membrane of Gram-positive sensitive bacteria, inhibit the synthesis of the cell wall, and, finally, lead to the cell death [49,50].

### 2.5. Cell Viability Assay

**Figure 7:**
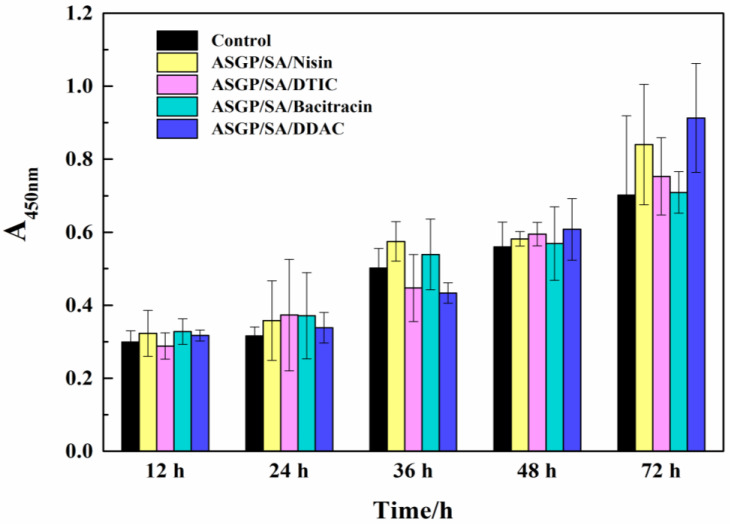
The cytotoxicity of the ASGP-SA-2/Nisin, ASGP-SA-2/DTIC, ASGP-SA-2/Bacitracin, and ASGP-SA-2/DDAC.

CCK-8 method was used to evaluate the biocompatibility of ASGP-SA-2/Nisin, ASGP-SA-2/DTIC, ASGP-SA-2/Bacitracin, and ASGP-SA-2/DDAC. RSC96 cells were inoculated on the hydrogel samples, and the proliferation of RSC96 cells in a certain culture time are shown in Figure 7. It was no toxicity for all the hydrogels to RSC96 cells after 12 h, and the OD values of the ASGP-SA-2/Nisin, ASGP-SA-2/DTIC, ASGP-SA-2/Bacitracin, and ASGP-SA-2/DDAC at 450 nm were 0.299 ± 0.031, 0.323 ± 0.063, 0.288 ± 0.036, 0.328 ± 0.035, and 0.317 ± 0.015, respectively. After 36 h of cell culture, OD values of cells in hydrogels were 0.502 ± 0.054, 0.575 ± 0.054, 0.447 ± 0.092, 0.539 ± 0.097, and 0.433 ± 0.028, respectively. The proliferation of RSC96 cells was more prominent in ASGP-SA-2/Nisin and ASGP-SA-2/Bacitracin gel. At 72 h, the OD values of cells in ASGP-SA-2/Nisin, ASGP-SA-2/DTIC, ASGP-SA-2/Bacitracin, and ASGP-SA-2/DDAC were 0.702 ± 0.217, 0.84 ± 0.165, 0.753 ± 0.106, 0.709 ± 0.057, and 0.913 ± 0.149, respectively. RSC96 cells showed more prominent proliferation in ASGP-SA-2/Nisin, and ASGP-SA-2/DDAC gels. CCK-8 results showed that hydrogels could support the adhesion and proliferation of RSC96 cells, especially ASGP-SA-2/Nisin hydrogels had better biocompatibility for RSC96 cells.

## 3. Conclusions

This study provides a possibility and significant insight into drug-loading bio-hydrogels with antibacterial activity based on ASGP natural materials cues performing a role in various biotechnology and tissue engineering fields. The antibacterial ASGP-SA drug-loaded hydrogels models had a swelling ratio above 60–70%, and more than above 80% cumulative drug release for 400 min. The ASGP-SA bio-hydrogels had more pro-proliferative effects on cells for 72 h. We further think of the optimizing ASGP-SA bio-hydrogels may securely be applied in other biomaterial field, such as targeting drug carriers and cell culture plat form in the future.

## 4. Materials and Methods

### 4.1. Materials

Fifth instar larvae of antheraea pernyi for preparation of regenerated ASGP were provided by the local farms (Dandong, China). Sodium alginate (SA, M*_W_* = 5.0 × 10^3^ KDa, Qingdao Bright moon Seaweed Group, Qingdao, China), metformin hydrochide (MFH, 97%, Shanghai Macklin Biochem Tech, Shanghai, China), RSC96 cellline (Guangzhou Jinio Biotechnology Co., Ltd., Guangzhou, China), CCK-8 kit (Cell Counting Kit-8, Biosharp White Shark Biotechnology Co., Ltd., Shanghai, China), cell culture medium (sterile filtration, Ge Healthcare Life Sciences Hyclone Co., Ltd., Beijing, China), Artificial blood (Dongguan Chuang feng Technology Co., Ltd., Dongguan, China). Nisin, 2,3-dihydroxypropyl trimethyl ammonium chloride (DTIC), bacitracin, dimethyldiallyl ammonium chloride (DDAC), and other materials and reagents were all purchased from Aladdin Bio-Chem Technology Co., Ltd., Shanghai, China.

### 4.2. Extraction of Antheraea pernyi Silk Gland Protein

Antheraea pernyi silk gland was obtained by dissecting the fifth instar larvae of antheraea pernyi. The above silk gland was cleaned three times with distilled water for removing the impurities on the surface of silk gland, then frozen at −30 °C for later use. The ASGP solution was obtained by dissolving above tussah silk glands (1.0 g) in mixed solvent of 1% SDS and Tris•HCl solutions (0.01 M, pH = 8.0) with shaking 2 h at room temperature, then centrifuged at 8000 rpm to achieve the ASGP solution.

### 4.3. Preparation of Drug Loaded Bio-Hydrogels

The ASGP solution was dissolved in the mixed solvent of 1% SDS and Tris•HCl solutions (0.01 M, pH = 8.0), then ASGP-SA mixed solution were prepared by added the 5% SA solution and the MFH drug model. The drug-carrying ASGP-SA mixed solution was poured into the curing solution containing CA curing agent for 24 h, then being freeze-drying (freezing 48 h, drying 48 h) to obtain drug-carrying bio-hydrogels with different compounding ratios. The specific preparation parameter of the drug-loaded hydrogels is shown in Table 3.

### 4.4. Preparation of Antibacterial Drug Loaded Bio-Hydrogels

Nisin, 2,3-dihydroxypropyl trimethyl ammonium chloride (DTIC), bacitracin, and dimethyldiallyl ammonium chloride (DDAC) were antibacterial agents and prepared 5% antibacterial solutions and refrigerated at 4 °C for later use. Considering the biodegradability and structural stability of drug loaded bio-hydrogels, the ASGP-SA-2 mixed solution was selected as a basis solution, then was added 10 mL 5% antibacterial solutions. The above mixed solutions were poured into 14% citric acid (CA) solution for 12 h, then freeze-dried to obtain the ASGP-SA-2/Nisin, ASGP-SA-2/DTIC, ASGP-SA-2/Bacitracin, and ASGP-SA-2/DDAC antibacterial drug loaded bio-hydrogels.

### 4.5. Morphology of Dried Drug-Loaded Hydrogel

The morphology of dried drug-loaded hydrogels samples after quenched in liquid nitrogen and sputtered with a thin layer of gold were observed by using a scanning electron microscope (JSM-IT100, JEOL, Tokyo, Japan).

### 4.6. FT-IR Analysis

The chemical structure of drug-loaded hydrogels was analyzed using a FT-IR spectroscopy (Nicolet IS10 FT-IR spectrometer, Thermo Fisher Scientific, Waltham, MA, USA) with the KBr technique at the wavelength range of 400–4000 cm^−1^ along with vacuum dryer for 4 h.

### 4.7. Swelling Ratio and Volume Stability Test

The dried drug-loaded hydrogels were removed top layer of hydrogels, then dried for 4 h at 40 °C. The swelling ratio and stability of above hydrogels were gravimetrically and volumetrically monitored by immersing the dried hydrogel samples (V_1_, W_1_) in water for 24 h. The mass of swollen hydrogels (V_2_, W_2_) was measured after removing the residual moisture. The experimental results were calculated from an average of three samples. The equilibrium swelling ratio (ESR) and volume stability (EVS) was defined as the weight and volume of absorbed water per weight of the dried hydrogel. The ESR and EVS were calculated as follows:

(4) $$\mathrm{ESR}\%=\frac{W_{2}-W_{1}}{W_{1}}\times 100\%$$

(5) $$\mathrm{EVS}=\frac{V_{2}-V_{1}}{V_{1}}\times 100\%\;\;\;$$

### 4.8. Anti-Bacterial Activity

The antibacterial drug carrier gels were evaluated by using inhibition zone test against the *S. aureus* (ATCC 25923) and *E. coli* (ATCC 25922). The hydrogel samples were cut into the diameters of 10~15 mm and reserved. The 10 mL nutrient agar medium (0.01% tryptone, 0.005% yeast extract, 0.01% sodium chloride, and 0.015% AGAR powder) was taken out and sterilized with autoclave at 121 °C for 20 min. The 0.1 mL overnight cultured bacteria solution was spread by a glass L-rod over the nutrient agar medium. Sterile tweezers were used to place the samples on the culture plate and incubated at standard condition culture (37 ± 2 °C, 18 h). The size of the inhibition zone was observed, and the inhibition zone was calculated, as shown in Formula (6). The test was done in triplicate.

H = (D − d)/2(6)

where H, D and d are the width bacteriostatic zone, the average outer diameter of bacteriostatic zone and the diameter of sample, respectively.

### 4.9. UV-Vis Assay

In order to simulate the human environment to study drug release behavior of drug-loaded hydrogels, artificial intestinal fluid simulation solution (SIF, pH = 7.4, 0.05 M KH_2_PO_4_, and 0.0395 M NaOH) was prepared as drug release medium. UV-Vis spectrophotometer (UV-T600, Beijing General Instrument Co. Ltd., Beijing, China) was used to evaluate the content of MFH in the drug-loaded hydrogels at 230 nm.

MFH standard curve is measured as followed: the release standard curve of MFH drug model in SIF was obtained by weighing 50 mg MFH and placing them in a 100 mL volumetric flask with 100 mL artificial intestinal fluid. The 2.5 ug/mL, 5.0 ug/mL, 7.5 ug/mL, 10 ug/mL, and 15 ug/mL MFH solutions were taken by diluting the above certain amount of MFH standard solution with SIF. UV-Vis spectrophotometer was introduced to obtain the absorbance of MFH solutions with different concentrations and blank contrast sample (SIF). The standard curve of MFH in SIF was achieved by taking the concentration of MFH as the abscissa, the absorbance of MFH as the ordinate.

Drug-loaded hydrogels drug release performance is measured as followed: The above drug-loaded hydrogels (150 mg) were putting in 100 mL SIF solutions at 37.0 ± 0.5 °C under magnetic stirring. The 5 mL above sample drug solution were taken out at the same interval (completed within 30 s), meanwhile, added the same amount of SIF. The absorbance of different time sample drug solution was measured at 230 nm. The cumulative release amount (Er), drug loading (D_L_) and encapsulation rate (E_N_) of the drug were determined according to metformin hydrochloride standard curve:

(7) $$E_{r}\left(\%\right)=\frac{C_{n}\cdot 100+5\sum_{i=0}^{n-1}C_{i}}{M_{0}}\times 100$$

(8) $$D_{L}\left(\mathrm{mg}/g\right)=\frac{M_{0}}{M_{1}}$$

(9) $$E_{N}\left(\%\right)=\frac{M_{0}}{M_{2}}\times 100\%$$

where C*_n_*_,_ C*_i_* is the concentration of the sample drug solution at the *n*th, *i*th time point; V_0_ = 0, C_0_ = 0. Er is one of cumulative release amount of the drug averages. M_0_ is the mass of the drug loaded into the drug-loading hydrogels. M_1_ is the total weight of loaded drug gel, M_2_ is the total weight of the drug input; each sample was tested for three groups and the average was calculated.

### 4.10. CCK-8 Assay

The biocompatibility of drug loaded hydrogels was evaluated by CCK-8 assay. RSC96 cells at logarithmic growth stage were cultured in a 96-well cell culture plate at 37 °C in a carbon dioxide incubator with 5% saturation for 3 days using cell suspension made from medium, serum, and dual antibodies. An amount of 100 uL RSC96 cell suspension was diluted and transplanted on the cell plate for 24 h, then a new cell culture medium was replaced. After incubation for 12 h, 24 h, 36 h, 48 h, and 72 h, the hydrogel sample and culture medium were removed. Cell culture medium was used as blank group. The same amount of CCK-8 culture mixture was added to the cell culture medium at different times mentioned above, and cultured in a CO_2_ incubator with 5% saturation at 37 °C for 30 min. The optical density was recorded at 450 nm with a microplate reader. The experiment was carried out three times. Cell toxicity was determined by cell survival rate, and cell survival rate was calculated according to Formula (10):

(10) $$C.V.=\frac{A_{sample}}{A_{control}}\times 100\%$$

where C.V. is cell survival rates; *A_sample_* is the OD value of sample extraction solution after cell culture, and *A_control_* is the OD value of the solution in the blank group.

## Figures and Tables

**Table 1:** The D_L_ and E_N_ of the drug loaded hydrogels.

Samples	E_N_ (%)	D_L_ (mg/g)
ASGP-SA-1	70.25 ± 1.05	450.3 ± 10.4
ASGP-SA-2	72.81 ± 1.23	432.5 ± 12.7
ASGP-SA-3	71.52 ± 1.20	425.3 ± 13.5
ASGP-SA-4	72.27 ± 1.52	402.5 ± 14.3

**Table 2:** Fitting parameters of three release dynamics models.

	First Order						Higuchi Model						Korsmeyer Peppas Model	
	R_1_^2^	k_1_	R_2_^2^	k_2_	R_3_^2^	k_3_	R_1_^2^	k_1_	R_2_^2^	k_2_	R_3_^2^	k_3_	R^2^	*n*
ASGP-SA-1	0.84	2.30	0.99	5.54	0.93	2.51	0.96	0.04	0.98	0.007	0.99	0.003	0.98	1.02
ASGP-SA-2	0.99	2.41	0.99	5.28	0.90	2.55	0.90	0.01	0.99	0.004	0.98	0.002	0.99	0.61
ASGP-SA-3	0.99	3.01	0.99	5.62	0.90	2.04	0.94	0.01	0.98	0.004	0.96	0.002	0.98	0.55
ASGP-SA-4	0.99	4.21	0.99	5.46	0.94	2.04	0.94	0.01	0.99	0.004	0.94	0.001	0.99	0.55

**Table 3:** The specific preparation parameters of drug loaded bio-hydrogels.

Sample	ASGP/(g/mL)	SA/(g/mL)	MFH/(g/mL)
ASGP-SA-1	2	8	0.15
ASGP-SA-2	4	6	0.15
ASGP-SA-3	6	4	0.15
ASGP-SA-4	8	2	0.15

## Data Availability

Data are contained within the article.
